# O04 Understanding the key role of accessory genes in AMR phenotype through interpretable machine learning techniques

**DOI:** 10.1093/jacamr/dlad066.004

**Published:** 2023-06-14

**Authors:** Lucy Dillon, Nicholas J Dimonaco, Christopher J Creevey

**Affiliations:** School of Biological Sciences, Queen’s University Belfast, BT7 1NN, UK; School of Biological Sciences, Queen’s University Belfast, BT7 1NN, UK; Department of Medicine, McMaster University, Hamilton, ON, Canada; School of Biological Sciences, Queen’s University Belfast, BT7 1NN, UK

## Abstract

**Background:**

Antimicrobial resistance (AMR) genes are found to be ubiquitous within the microbiome, even when antimicrobial usage is absent. To identify the AMR phenotype, the most common method is to use a laboratory-based assay. Yet, when dealing with samples from the microbiome, many species are difficult to culture within the laboratory. The vast quantity of strains would be time-consuming to culture. To avoid this, a computational approach may be a more favourable choice. AMR gene finder tools are efficient at determining the AMR genotype. Despite this, how a genotype relates to the AMR phenotype is still an open question.

**Methods:**

To evaluate the relationship between the AMR phenotype and the AMR genotype, 16 950 genomes from BV-BRC which had corresponding MIC values were analysed. Using Weka’s J48 decision tree model, the relationship between the AMR phenotype and the AMR genotype was analysed. The role of accessory genes in relation to the AMR phenotype was analysed in the same way.

**Results:**

The J48 models could predict the AMR phenotype accurately using AMR genes and accessory genes; the average accuracy was 91.7% and 92.2%, respectively. The results found that gene co-occurrence, presence and absence of genes are key factors to analyse when identifying the AMR phenotype from genomic data. These factors are not evaluated by commonly used AMR gene finder tools, which could miss vital information to determine the correct phenotype.

**Conclusions:**

Our results highlight why we should continue to research the relationship between the AMR phenotype and genomic data.

